# On the Role of Hydrogen Bond Strength and Charge Transfer in an On‐Water Diels–Alder Reaction: Semiempirical and Free Energy Calculations

**DOI:** 10.1002/jcc.70467

**Published:** 2026-07-20

**Authors:** Andrés Henao, Yomna Gohar, René Wilhelm, Thomas D. Kühne

**Affiliations:** ^1^ Dynamics of Condensed Matter, Chair of Theoretical Chemistry Paderborn University Paderborn Germany; ^2^ Institute of Organic Chemistry, Clausthal University of Technology Clausthal‐Zellerfeld Germany; ^3^ Center for Advanced Systems Understanding (CASUS) Görlitz Germany; ^4^ Helmholtz Zentrum Dresden‐Rossendorf Dresden Germany; ^5^ Institute of Artificial Intelligence, Technische Universität Dresden Dresden Germany

**Keywords:** charge transfer, density‐functional tight binding, Diels–Alder reaction, free energy calculations, hydrogen bonding, on‐water catalysis

## Abstract

On‐water catalysis is widely exploited, yet the microscopic origin of rate enhancements at aqueous interfaces remains debated. In the spirit of the second‐generation Car‐Parrinello method, we use a modified Langevin‐type scheme to accelerate density‐functional based tight‐binding molecular dynamics in order to investigate the free energy of two Diels‐Alder reactions on‐water: the cycloaddition of cyclopentadiene with ethyl cinnamate and that with ethyl thionocinnamate. The only difference between the two studied molecules is that the carbonyl oxygen of the cinnamate is a thiocarbonyl sulfur in the thionocinnamate; hence, the first is a strong and the second a weak hydrogen bond acceptor. The oxygen‐containing dienophile reacts through an almost synchronous transition state with a barrier of ~13 kcal/mol, whereas the sulfur analogue follows an asynchronous pathway with a barrier of ~24 kcal/mol because a transient S—C contact competes with C—C bond formation. We find no increase in the number or strength of hydrogen bonds at the transition state. Instead, the catalytic role of the interface is to maintain an electronically productive hydrogen‐bond environment that stabilizes charge reorganization along the reaction coordinate. These results indicate that, for the present on‐water reactions, hydrogen‐bond quality and charge‐transfer capability are more decisive than the hydrogen‐bond count alone.

## Graphical abstract

Matched Diels‐Alder dienophiles at a water interface reveal that on‐water catalysis depends on hydrogen‐bond quality rather than hydrogen‐bond count. Oxygen preserves charge‐transfer coupling and lowers the free‐energy barrier, whereas sulfur disrupts this coupling and promotes an asynchronous pathway.

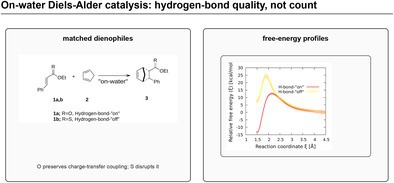

**Abbreviations**
CPD: cyclopentadiene
DFTB: density‐functional based tight binding
SCF: self‐consistent field
WHAM: weighted histogram analysis method

## 1 Introduction

Water is not only a bulk solvent of biological matter, but also an active component of enzyme catalysis: ordered water molecules and hydrogen‐bond networks in active sites can relay protons, tune local electrostatics, and stabilize transition states. The central importance of hydrogen bonding in condensed phases has been recognized since early spectroscopic and physical–chemical studies by Errera and Mollet and by Wolf, Prahm, and Harms [1, 2]. However, its use in chemistry is less established, as many substances of interest are not soluble in water, which has restricted the use of water for chemical reactions. The discovery of the acceleration of Diels‐Alder reactions of small nonpolar groups dissolved in water in 1980 [3], and the more recent catalysis on‐water [4], has changed this panorama. Lately, more examples of the acceleration of chemical processes at the interface with water have been described [5, 6, 7, 8, 9].

The same chemical principle also underlies the broad field of hydrogen‐bond‐donor organocatalysis. Explicit hydrogen bonding by small organic catalysts, including urea, thiourea, squaramide, and anion‐binding catalysts, is now an established activation mode rather than an isolated motif [10, 11, 12, 13, 14, 15]. For this reason, on‐water catalysis is best discussed together with the wider question of when a hydrogen‐bond donor environment promotes a reaction by contact formation alone and when it also controls polarization and charge reorganization.

The mechanism by which reactions are accelerated at aqueous interfaces is still not clear due to two main reasons [16]. Firstly, a theory of solvation at interfaces is more complex than bulk solvation and is an active topic of research. Secondly, the wide variety of phenomena occurring at aqueous interfaces may imply that there is a variety of mechanisms, making the interpretation of experimental results a complex task. Computational models are highly desirable to understand experimental results and elucidate the underlying mechanisms driving the reactions at aqueous interfaces. Jung and Marcus proposed a mechanism for on‐water catalysis based on the availability of free OH bonds at the interface, interacting through hydrogen bonds with the molecular species [17]. This is in contrast to the homogeneous in‐water case, where hydrogen bonds require higher energy to be broken and are therefore less available [3]. Yet, in‐water reactions can be accelerated due to the Breslow hydrophobic effect [18, 19]. Other mechanisms for the acceleration of on‐water reactions have also been proposed. Recently, an experimental study found an additional increase in the reaction rate of diethyl azodicarboxylate and quadricyclane cycloaddition when performed on micro‐droplets, which they coined “on‐droplet” chemistry [20]. It has been suggested that this is due to the surface‐to‐volume ratio and supports the acid catalysis mechanism of Beattie and co‐workers [21], where reaction with water at the interface results in both the protonated substrate and free OH^−^, which is stabilized by its strong adsorption at the interface. Interestingly, a recent review presents contradictory and unexplained mechanisms. Instead, a revised on‐water model is proposed [22], where the interfacial water molecules would undergo partial polarization because of a loss of balance between donation and acceptance of hydrogen bonding through the Grotthuss mechanism at the interface.

The wide variety of proposed mechanisms for the acceleration of reactions at the interface with water, highlights the importance of using computational methods to shed light on the catalysis mechanism at water interfaces. The studied systems have been, however, limited by the high computational cost of quantum mechanical electronic structure calculations. The use of improved methods allows us to treat the required degrees of freedom efficiently and at the same time accurately, which allows us to analyze the reactions at realistic heterogeneous interfaces. This is of great importance in order to uncover the interplay of hydrogen bonds and reaction accelerations on‐water. For closely related Diels–Alder chemistry, Jorgensen and co‐workers already used Monte Carlo free‐energy simulations to study the cycloaddition of cyclopentadiene with methyl vinyl ketone in water [23, 24]. Those pioneering calculations established that water can accelerate such reactions through preferential hydrogen bonding and solvent reorganization around a polarized transition state. The present work is a natural extension of that idea, but differs in three essential respects: we sample an explicit heterogeneous water interface rather than homogeneous bulk solution, compute free‐energy profiles from periodic molecular dynamics and umbrella sampling rather than equilibrium Monte Carlo perturbation, and use a matched carbonyl/thiocarbonyl pair to isolate the role of hydrogen‐bond acceptor strength. Previous on‐water reaction studies have shown some insights. A lowering of the free energy barrier has been reported for a solvated retro‐Diels‐Alder reaction using 50 water molecules (in‐water) in comparison to the vapor phase reaction. The authors used Car‐Parrinello molecular dynamics simulations and concluded that the water solvent is pivotal for this effect, especially for compounds containing an electron‐donating substituent [25]. The role of microsolvation on reaction rates has also been highlighted. Pestana et al. used ab initio simulations to investigate a retro‐Diels‐Alder reaction in bulk water under different confinements and found that the acceleration was similar between the different confinements [19]. Therefore, the determining factor for the catalysis is the microsolvation, which implies that no additional catalysis would be expected for on‐water via a local hydrogen‐bond mechanism. Karhan et al. compared the homogeneous with heterogeneous Diels‐Alder reaction between dimethyl azodicarboxylate (DMAD) and quadricyclane (Q) [26], and found that the number of hydrogen bonds for the transition state and reactants was similar and only slightly increased as expected from Jung and Marcus [17]. Recently, Salem and Kühne revisited the DMAD with Q reaction using an energy decomposition analysis of the reactants, the transition state and the product in the presence of three water molecules, and discovered that charge transfer plays an important role [27]. Yang and co‐workers also recently highlighted the role of charge transfer using an ab initio quality neural network to model a cycloaddition reaction on‐water [28].

**FIGURE 1:**
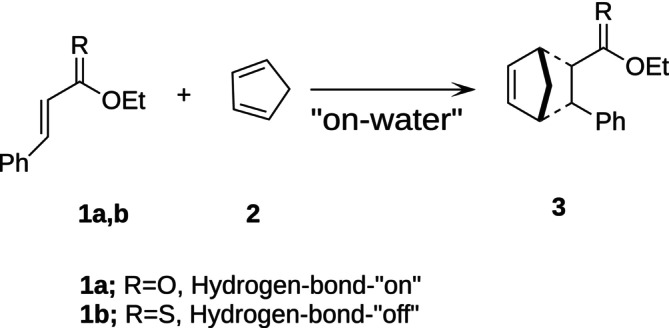
Diels‐Alder reaction with cinnamates (1) and cyclopentadiene (2) at the interface with water. The cinnamate is shown in the anti (*E*) arrangement of the phenyl and (thio)carbonyl substituents. When the carbonyl oxygen in ethyl cinnamate (1a, or hydrogen‐bond‐“on”) is replaced by a thiocarbonyl sulfur, a thionocinnamate (1b, or hydrogen‐bond‐“off”) is obtained. The product 3 shows the newly formed bonds (dashed lines) between 1 and 2. The average bond distance of these two newly formed bonds will be used as a reaction coordinate for our free energy calculations.

In this study, we investigate the importance of hydrogen bond strength for accelerating an on‐water Diels‐Alder reaction, inspired by an experimental study on the Diels‐Alder reaction with ethyl crotonthioate as dienophile [29]. We studied two systems, shown in Figure 1, of two slightly different reactants that can both form bonds with cyclopentadiene. The first reactant is ethyl cinnamate, which contains a carbonyl oxygen, which we will refer to as the H‐bond‐“on” system from now on. The second reactant is ethyl thionocinnamate [29], hence a thiocarbonyl sulfur replaces the carbonyl oxygen. As sulfur has a weaker hydrogen‐bond interaction with water [29], we will call this the H‐bond‐“off” system. Direct kinetic data for this exact matched pair under the same interfacial conditions are not available to us; the computed barrier difference should therefore be read as a mechanistic and experimentally testable prediction, anchored in the known behavior of related sulfur‐containing dienophiles.

## 2 Computational Details

The modeling of a realistic heterogeneous interface is an important aspect in order to investigate the effect of the water interface on the acceleration of reactions. To achieve this, we have used the second‐order density functional tight‐binding (DFTB2) approach [30], as implemented in the CP2K suite of programs [31, 32]. This method allows us to approximately treat the quantum nature of the electrons by determining the charge density self‐consistently. The efficiency of DFTB2 lies in between density functional theory and classical molecular dynamics. Even though systems in the order of thousands of atoms are now feasible with DFTB2 [33, 34], the evaluation of the free energy of the chemical reactions at the interface requires several simulations, sampling at different points along a reaction coordinate. To make this possible, we employ a grand‐canonical linear‐scaling technique together with the second‐generation Car‐Parrinello method [35, 36], where the numerical precision is reduced [37], and a modified Langevin scheme is used to compensate for this [38], thereby improving the efficiency [39], while keeping the accuracy of the self‐consistent charge densities [33]. This allowed us to efficiently perform more than 70 simulations for the two systems of interest and to obtain the free energy profile of the on‐water reactions.

A CP2K‐specific aspect of this setup is that the semiempirical Hamiltonian is embedded in the same periodic simulation infrastructure used by the other electronic‐structure methods in CP2K. The short‐ranged Slater–Koster Hamiltonian and overlap matrices, as well as the repulsive pair interactions, are assembled from sparse neighbor‐list blocks over the periodically repeated cell. In self‐consistent‐charge DFTB, the short‐ranged $\gamma_{\mathrm{AB}}\left(R\right)$ interaction is complemented by CP2K's tight‐binding smooth particle‐mesh Ewald treatment of the long‐ranged 1/R contribution, including the corresponding self and neutralizing‐background terms. The same implementation accumulates consistent force and virial contributions from the Hamiltonian, overlap, repulsive, and electrostatic terms. This is the CP2K ingredient that makes the DFTB2 dynamics used here directly compatible with large heterogeneous periodic interface simulations.

Each system consisted of 22 cyclopentadiene molecules, 4 cinnamates, and 278 water molecules. This composition was chosen as a compromise between interfacial realism and the need to run many umbrella windows: the water content forms a stable slab with two liquid–vapor interfaces under periodic boundary conditions, four cinnamate molecules provide multiple independent interfacial acceptors without creating an organic phase, and the excess cyclopentadiene keeps the diene available at the interface throughout the sampling. The reactant molecules were chosen close to the interface with water, and they remained there during the simulation time. We used the original mio‐1‐1 parameters for the organic molecules [30], as well as modified force‐matched parameters for liquid water (mio‐0‐1 + BI) [40]. The mio‐0‐1 + BI parameters improved the O—O and O—H repulsive potentials using a reference higher theory level calculation using a Car‐Parrinello simulation of 32 water molecules using the PBE functional [30].

Free energy calculations were done using an umbrella sampling potential $k{\left(\xi -\xi_{0}\right)}^{2}$ along with the reaction coordinate ξ [41]. This reaction coordinate was chosen as the average distance of the two bonds that are formed in the Diels‐Alder reaction (Figure 1). The free energy was evaluated along this coordinate between 4.5 Å (the reactants) and 1.5 Å (the product) at every 0.1 Å with a force constant of k=470 kcal/mol/Å^2^. Four additional umbrella windows were simulated close to the identified transition states with a tighter force constant of k=1880 kcal/mol/Å^2^ and at every 0.05 Å, simulating two additional umbrella windows at each side of the transition state. The weighted histogram analysis method (WHAM) was employed to recover the eventual unbiased free energy profiles [42, 43].

## 3 Results

**FIGURE 2:**
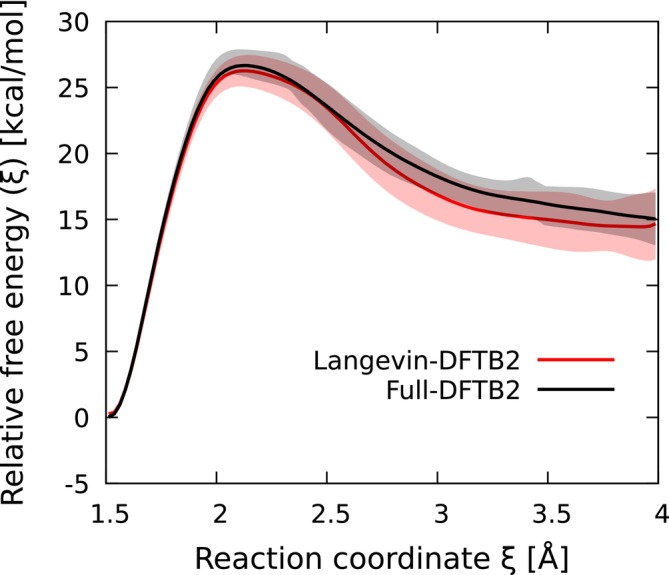
Relative free energy profiles as obtained by the full‐DFTB2 (black curve) and the accelerated Langevin‐DFTB2 methods (red curve), respectively. The umbrella sampling simulations were analyzed using the WHAM code [43]. The standard deviations were calculated using the bootstrap analysis and shown as a shaded areas behind each curve. These results show that the Langevin‐DFTB2 method entails free energy profiles that are identical (within the statistical uncertainty) to those obtained by the full‐DFTB2 method, though at reduced computational cost.

The use of an approximate linear‐scaling method along with the DFTB2 method was first tested to assess its accuracy in obtaining free energy profiles. For this, the converged full‐DFTB2 reference free energy profile was obtained using explicit diagonalization in conjunction with a tight self‐consistent field (SCF) threshold of $10^{-7}$, and compared to a set of equally long second‐generation Car‐Parrinello simulations using the modified Langevin approach with a moderate SCF criterion of $10^{-6}$, resulting in a 4‐fold increased efficiency for the Langevin‐DFTB2 scheme. In Figure 2, the relative free energies, which are identical within the corresponding statistical uncertainty (error sampling was obtained using the bootstrap analysis implemented in the WHAM code [43]), are compared to each other, thereby indicating that the Langevin‐DFTB2 method provides accurate estimates of free energies. Therewith, we employed Langevin‐DFTB2 to obtain 100 ps simulations for each of the 35 different umbrella windows along ξ for both the H‐bond‐“on” and the H‐bond‐“off” systems.

**FIGURE 3:**
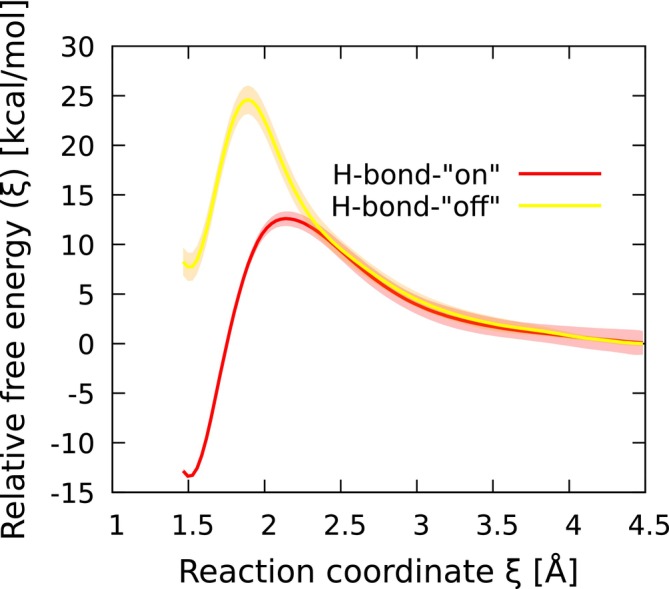
Relative free energy profiles for the cycloaddition of cyclopentadiene with ethyl cinnamate (H‐bond‐“on”) and ethyl thionocinnamate (H‐bond‐“off”) at the water interface, obtained from umbrella sampling simulations using the accelerated Langevin‐DFTB2 scheme for the H‐bond‐“on” (red curve) and H‐bond‐“off” (yellow curve) systems, respectively. The shaded area shows the statistical errors as estimated using the bootstrap analysis within the WHAM code [43].

The resulting relative free energy profiles are shown in Figure 3 for both the H‐bond‐“on” (red curve) and ‐“off” (yellow curve) systems. The different shape of the two free energy profiles indicates a different activation of the cycloaddition reaction even though the two systems differ only by the carbonyl C=O or thiocarbonyl C=S group, which do not directly participate in the formation of new chemical bonds. The energy barrier of formation is obtained as the difference in the relative free energies between the reactants (at the ξ≈4.5 Å) and the transition state. In the case of the H‐bond‐“on” system, the transition state is located at ~2.1 Å and the energy barrier is ~13 kcal/mol, while the transition state for the H‐bond‐“off” system is located around ~1.9 Å and the energy barrier is ~24 kcal/mol, respectively. The resulting difference of about 11 kcal/mol is chemically substantial: if translated into a rate effect through transition‐state theory, it would correspond to many orders of magnitude at room temperature. Thus, the replacement of oxygen by sulfur does not merely perturb a passive substituent; it changes the interfacial electronic response of the dienophile in a way that is directly reflected in the free‐energy barrier. Beside the larger free energy barrier for the Diels‐Alder reaction occurring at the water interface of the H‐bond‐“off” system, another difference between the two reactions is the free energy of formation of the products, that is, $\Delta A=A_{\text{product}}-A_{\text{reactant}}$. This is ΔA<0 (−14 kcal/mol) for the H‐bond‐“on” system and ΔA>0 (+7 kcal/mol) for the H‐bond‐“off” system, indicating an exothermic and endothermic reaction, respectively. Next, the umbrella sampling trajectories were analyzed to obtain atomistic insights of the differences in the free energy barriers and corresponding energies of formation.

**FIGURE 4:**
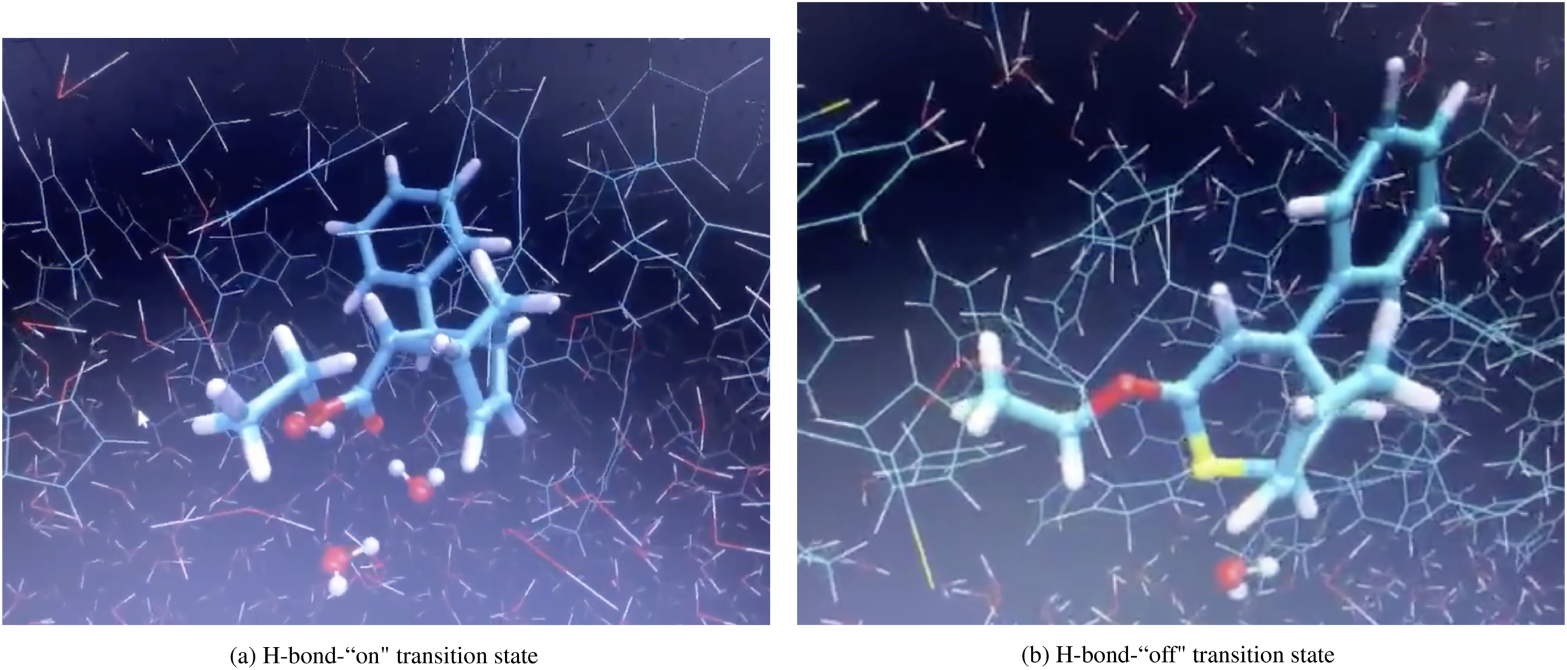
Snapshot of the transition states of the H‐bond‐“on” and H‐bond‐“off” systems, respectively. The formation of the second C—C bond is more stable for the H‐bond‐“on” system. The transition state of the H‐bond‐“off” system reveals the transient formation of a further S—C bond between the thionocinnamate and cyclopentadiene. This competition against the formation of the second C—C bond is responsible for the different relative free energy profile and higher energy barrier shown in Figure 3. Figure obtained using VMD [44].

A snapshot of the trajectories for each umbrella sampling simulation at the window corresponding to the transition states, at ~2.1 Å for the H‐bond‐“on” and ~1.9 Å for the H‐bond‐“off” system is shown in Figure 4. A representative snapshot of the transition state of both systems revealed that the formation of the two C—C bonds occurs in an asynchronous way, that is, the two C—C bonds are not simultaneously formed. For both systems, the shorter and therefore earlier‐forming bond connects cyclopentadiene with the β carbon of the cinnamate double bond, that is, the carbon farther from the (thio)carbonyl group and bonded to the phenyl substituent; the second bond involves the α carbon adjacent to the (thio)carbonyl group (see Figure 1). However, the second C—C bond is formed in the H‐bond‐“on” system relatively easily, as it occurs in a single step. This is not the case for the H‐bond‐“off” system, where a transient bond is observed in Figure 4b between the sulfur and a carbon atom of cyclopentadiene [45, 46]. This implies that the formation of the second C—C bond has to overcome an additional energy barrier in the H‐bond‐“off” system due to an interaction between the sulfur and cyclopentadiene. Thus, it is revealed that the carbonyl C=O species plays a stabilizing role during the approach of the reactants at the interface with water, and that the thiocarbonyl C=S species interferes with the formation of the second C—C bond which can be attributed to the stronger electronegativity of oxygen compared to sulfur and its ability to form stronger hydrogen bonds with water. However, further analyses are necessary to understand this mechanism in detail. Next, we have quantified the asynchronicity of the transition state as a way to characterize the mechanism of the Diels‐Alder reaction by defining it as the difference in length between the newly formed bonds at the transition state, that is, $\Delta {C=C}_{\text{bond}}^{1}-C_{\text{bond}}^{2}$. The value of ΔC is small for the H‐bond‐“on” system and large for the H‐bond‐“off” system, respectively. Specifically, $\Delta C_{“\mathrm{on}”}=0.02\pm 0.20$ Å and $\Delta C_{“\mathrm{off}”}=0.95\pm 0.07$ Å, which indicates that the reaction happens synchronously in the strong H‐bonded system and that it is asynchronous in the weak H‐bonded system. This supports the notion that different reaction mechanisms are at work between these two systems, as indicated by the different shapes of the free energy profiles in Figure 3.

The bond formation can be qualitatively traced using a charge population analysis at different steps during the reaction. Due to the ability of DFTB2 to self‐consistently redistribute the electronic charges, the popular Mulliken population analysis is employed in this work [47]. While there are other schemes, such as the density derived electrostatic and chemical net atomic charges [48, 49], Mulliken charges provide a sufficiently accurate qualitative picture, when considering the approximations already made by the choice of the DFTB2 method. Applying a reweighting to the biased charges $P^{\prime }\left(q\right)$, we recovered the corresponding unbiased charges via

(1) $$P\left(q\right)\propto P^{\prime }\left(q\right)\exp\left(\frac{k{\left(\xi -\xi_{0}\right)}^{2}}{k_{B}T}\right).$$

**FIGURE 5:**
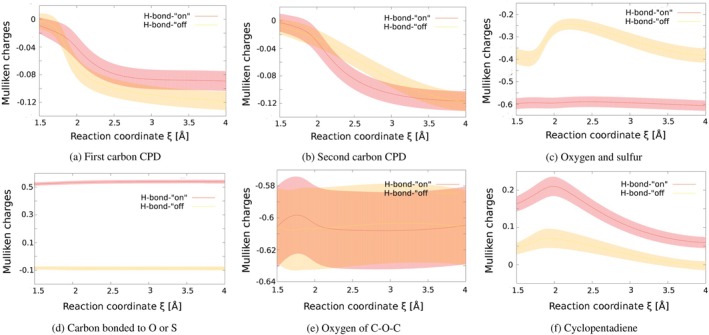
Mulliken charges for different atoms of the H‐bond‐“on” (red curves) and H‐bond‐“off” (yellow curves) systems, respectively.

We then analyzed the charge population for six different groups shown in Figure 5. First, the two carbon atoms directly involved in the cycloaddition reaction (Figure 5a,b) show a different charge population evolution on the second carbon atom for the H‐bond‐“off” system, which is consistent with the earlier shown trajectories and asynchronicity of the transition states. Figure 5c shows a large difference in the Mulliken charges between the carbonyl oxygen and thiocarbonyl sulfur atoms. The oxygen atom has a constant charge along the reaction path, whereas sulfur obeys a decrease of charge around the transition state. This is in line with the transient bond formation observed in the transition state trajectories between the sulfur and cyclopentadiene. Also, oxygen is more electronegative (∼−0.6e) than sulfur (∼−0.4e), which makes it a better hydrogen bond acceptor for interfacial water. The charge of the carbon atom bonded to this oxygen or sulfur atom is presented in Figure 5d, and showing no change along the reaction path for either system. The same is true for Figure 5e, which shows the oxygen atom in the C—O—C group of ethyl cinnamate or ethyl thionocinnamate that has a constant charge in both systems. Finally, the total charge of cyclopentadiene, shown in Figure 5f, increases from reactant to product and has a maximum that corresponds with the position of the transition state in the free energy profile of Figure 3. The change in the total charge of cyclopentadiene is larger for the H‐bond‐“on” system. This larger charge response is consistent with a more efficient polarization of the reacting pair by the hydrogen‐bonded carbonyl group, whereas the sulfur‐containing analogue diverts part of the electronic reorganization into a competing S–C interaction. Next, we investigated the role of hydrogen bonds along the reaction profile.

**FIGURE 6:**
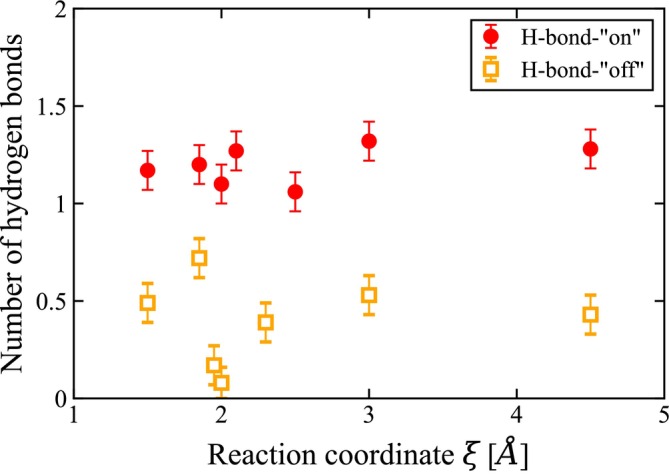
Mean number of hydrogen bonds between water molecules and the carbonyl (C=O) oxygen and the thiocarbonyl (C=S) sulfur atom along our reaction coordinate. Each point is a WHAM‐reweighted ensemble average over a binary hydrogen‐bond criterion, so noninteger values denote population averages rather than fractional bonds. The red (upper) symbols show the results for the H‐bond‐“on” system. The number of hydrogen bonds remains constant within error bars along the reaction path. The yellow (lower) symbols show the corresponding results for the H‐bond‐“off” system. There is a slight decrease in the number of hydrogen bonds around the transition state.

An H‐bond increase at the transition state has been suggested as a mechanism to explain the increase of reactivity on‐water [17]. The Mulliken charges of oxygen and sulfur showed the largest difference in the charge evolution along the reaction path (Figure 5c) and in addition, the sulfur atom was found to behave differently and unexpectedly bond to cyclopentadiene at the transition state (Figure 4b). We therefore computed the number of hydrogen bonds between the water molecules and the carbonyl (C=O) oxygen, as well as the thiocarbonyl (C=S) sulfur atoms. In fact, the hydrogen bonding to water by the reactants occurs through these atoms only. We applied the same weight shown in Equation (1) to obtain the unbiased number of hydrogen bonds. The reported hydrogen‐bond number is an ensemble average of a binary geometric hydrogen‐bond indicator over the reweighted umbrella ensemble, not the number of bonds in one selected configuration. Consequently, a value such as 1.5 means that one contact is nearly persistent and an additional contact is present in roughly half of the sampled configurations; it does not imply a fractional chemical bond. Our results (Figure 6) show different trends for the number of hydrogen bonds for the two systems. In the case of the H‐bond‐“on” reaction, the number of hydrogen bonds is almost constant (within statistical uncertainty) and ∼1.2, while the number of hydrogen bonds clearly changes along the reaction path for the H‐bond‐“off” system. The small nonmonotonic variations of the H‐bond‐“on” curve, including the apparent decrease between ξ≈3.0 and 2.0 Å, are within the statistical uncertainty and reflect interfacial orientation and solvent‐accessibility fluctuations rather than a systematic weakening of the carbonyl acceptor. It decreases to nearly zero around the transition state (1.9 Å), followed by an increase at 1.8 Å, before finally reaching a value of ∼0.5 for the product, which is similar to that of the reactants. The comparison with Figure 5c also shows that the H‐bond population is not determined by the Mulliken charge alone. For oxygen, the charge remains nearly constant and the H‐bond population changes only weakly; for sulfur, the electronic change near the transition state coincides with the loss of water contacts because the same sulfur atom becomes involved in the transient S–C interaction. This is in accordance with the trajectories of the transition states we observed, where the sulfur is also bonded to cyclopentadiene and competes with the formation of the second C—C bond. Additionally, we analyzed the distribution of hydrogen bond distances and angles for both systems, as shown in Figures S1 and S2, respectively, but found no evidence for shorter hydrogen bonds in the transition state.

## 4 Discussion

Contrary to the current understanding of the H‐bond mechanism [17, 50], we have not observed an increase in the number of hydrogen bonds (Figure 6) at the transition state of the system with a stronger H‐bond interaction (the H‐bond‐“on” system). In the case of the system with a weaker H‐bond interaction (the H‐bond‐“off” system), the number of hydrogen bonds even decreased at the transition state. This decrease coincides with the increase in the activation energy of the reaction and is linked to the competition of C—C bonding with the interaction between the sulfur atom and cyclopentadiene. At the molecular level, the weak H‐bond acceptor therefore does not simply remove one stabilizing contact with water. Instead, it opens an alternative soft sulfur–carbon interaction that distorts the approach geometry of the dienophile and delays the formation of the second C—C bond. The consequence is an additional free‐energy cost for the cycloaddition reaction of the H‐bond‐“off” system. We propose that the role of hydrogen bonds, and therefore the role of the water interface, is to stabilize the configurations of the reactants in order that the formation of new bonds may occur.

Our results show that the system with a weaker H‐bond interaction (as sulfur has a lower electronegativity compared to oxygen) has to overcome a larger energy penalty caused by the changing conformation of the reactants by interacting via the thiocarbonyl sulfur atom. We have also not found any indication of strengthened hydrogen bonds at the transition states compared to at the reactants or products. Our hypothesis is that the presence of hydrogen bonds is important as there is a higher penalty for the H‐bond‐“off” reaction (11 kcal/mol compared to H‐bond‐“on” reaction). In the H‐bond‐“off” system, we observed a depletion in the number of hydrogen bonds around the transition state (Figure 6).

This interpretation is closely related to the energy‐decomposition analysis of Salem and Kühne [27], who showed that hydrogen bonding can mediate on‐water catalysis through electrostatic, polarization, and charge‐transfer stabilization rather than through a purely structural contact count. The present free‐energy simulations extend that picture from a few‐water cluster model to a fluctuating heterogeneous interface. In our trajectories, the decisive quantity is not the maximum number of H‐bonds at the transition state, but whether the interfacial H‐bond network can keep the reactive carbonyl group electronically coupled to water while the C—C bonds form. For the oxygen‐containing dienophile this coupling is maintained; for the sulfur‐containing analogue it is partially lost and replaced by a competing interaction with cyclopentadiene. Thus, Reference [27] and the present work point to the same chemical conclusion: interfacial hydrogen bonds are catalytically relevant when they enable charge reorganization along the reaction coordinate.

QM/MM studies of the reaction of cyclopentadiene (CPD) with methyl vinyl ketone (MVK) in water suggested that the H‐bond is enhanced at the transition states, observing a shortening of the H‐bond distance and the influence of water reorientation [51, 52]. We found however, no evidence of a shortening of hydrogen bonds at the transition state (see Figures S1 and S2) for the Diels‐Alder reaction of this work at the interface with water, that is, on‐water. A similar study using QM/MM molecular dynamics showed an acceleration of the same reaction in water compared to methanol [53]. The authors assigned responsibility for the acceleration to the H‐bond formed between the carbonyl oxygen atom and hydrogen atoms in the solvent at the transition state, which polarized the C=O bond and stabilized the system charge reorganization during the reaction. We found a similar mechanism after analyzing the charge population (Figure 5), even though our results are from at the interface instead of the bulk, this suggests that there is a stabilization of the transition states through a charge redistribution which contributes to acceleration of the reactions. The role of charge transfer has also been discussed by other authors [27, 53]. Recent studies of the water/oil interface showed significant buildup of surface charge density at hydrophobic interfaces [54] and the importance of surface electric fields as a source of microdroplet reactivity [55]. In this broader context, the present results support a chemically nuanced view of on‐water catalysis: the interface can preorganize reactants, provide directional H‐bond donors, and polarize the reacting complex, but these effects need not appear as a larger number of shorter H‐bonds at the transition state. They may instead appear as a different electronic pathway, as seen here in the charge evolution of cyclopentadiene and in the sulfur‐induced asynchronous mechanism. This is, however, not a general statement, as we have studied a single reaction and the wide variety of phenomena occurring at aqueous interfaces may imply different mechanisms for other reactions.

The thermodynamic consequences are also consistent with earlier suggestions that interfacial reactions can profit from preorganization and reduced configurational entropy penalties [56, 57]. We have not decomposed the free energy into enthalpic and entropic contributions here, but the different mechanisms seen for the two substrates suggest that interfacial preorganization alone is not sufficient. A productive on‐water reaction also requires that the preorganized geometry remains electronically productive as the transition state is approached. This provides a practical criterion for future reaction design and for the selection or design of hydrogen‐bond donor catalysts: functional groups that are good H‐bond acceptors and remain electronically coupled to the water interface should be favored, whereas softer or more polarizable groups may introduce competing contacts that slow or redirect the cycloaddition. In practical terms, this suggests screening dienophiles and catalyst environments not only for the number of possible H‐bonds, but also for whether those H‐bonds preserve charge‐transfer coupling as the C—C bonds form. Such descriptors could guide the choice of hydrogen‐bond donor motifs in established organocatalyst classes and help identify reactant/catalyst combinations in which interfacial or catalyst‐bound water remains electronically productive.

Future calculations could test this criterion by combining the present umbrella‐sampling strategy with ALMO or related energy‐decomposition analyses along the reaction coordinate, thereby separating electrostatics, polarization, and charge transfer at the actual interface. It would also be useful to follow a substituent series between carbonyl and thiocarbonyl dienophiles, and to quantify how imposed interfacial electric fields, surfactants, or droplet curvature change the same charge‐transfer descriptors. Such calculations would connect the molecular mechanism identified here to the broader phenomenology of on‐water and on‐droplet chemistry [16, 20, 22, 55].

## 5 Conclusions

We have studied the Diels–Alder reaction between cyclopentadiene and two cinnamates occurring at the water interface using density‐functional based tight‐binding (DFTB2 [30]) molecular dynamics. The modeling of a realistic interface was made possible by using an acceleration scheme in the spirit of the second‐generation Car‐Parrinello method [35]. The role of the hydrogen bond in the enhancement of the reactivity on‐water was investigated by comparing the free energy barrier of the reaction for the two systems, one which is a strong hydrogen‐bond acceptor (ethyl cinnamate) and a second weak hydrogen‐bond acceptor (ethyl thionocinnamate).

The strong hydrogen‐bond acceptor reacts through nearly synchronous C—C bond formation, whereas the weak hydrogen‐bond acceptor follows an asynchronous path in which a transient S–C contact competes with cycloaddition. According to our simulations, the role of hydrogen bonds at the water interface for the systems here studied is the stabilization of the conformations of the reactants. Charge transfer stabilization of the transition states was also observed in our simulations. These results are in agreement with previous studies suggesting the importance of charge reorganization and polarization in the transition states in the presence of water [27, 53] and the buildup of surface charge at water/oil interfaces [54, 55]. Regarding the role of hydrogen bonds, we found no evidence for the increase in the number of hydrogen bonds (with respect to the reactants or products), nor strengthened hydrogen bonds at the transition states as suggested by Jung and Marcus [17]. The free energy barrier of the reaction for the strong hydrogen‐bond acceptor system was lower when compared to the weak hydrogen‐bond system. A correlation was also observed with the decrease in the number of hydrogen bonds at the transition state for the weak H‐bond system. The difference in hydrogen bonding is also observed as a variation of the charge of the sulfur atom at the transition state while the charge of the oxygen atom is constant for the complete reaction profile. Together, these observations indicate that hydrogen‐bond strength, electronic coupling to the interface, and charge‐transfer capability are more important than the number of hydrogen bonds alone. For on‐water catalysis, the main implication is that the interface should be viewed as an electronically active environment rather than as a simple source of additional H‐bonds. Productive catalysis requires a match between interfacial preorganization, H‐bond acceptor strength, and charge‐transfer capability of the reacting substrate. This conclusion should nevertheless not be read as universal, since different classes of aqueous interfaces and substrates may exploit different combinations of preorganization, polarization, proton transfer, and charge transfer.

## Funding

This work was supported by the European Research Council (ERC) (716142), Deutsche Forschungsgemeinschaft (DFG) (417590517/CRC1415 and 519869949).

## Supporting information


**Figure S1:** WHAM‐reweighted hydrogen‐bond distance and angle distributions for water contacts to the carbonyl oxygen in the H‐bond‐“on” system. The distributions compare representative reactant, transition‐state, and product regions along the same reaction coordinate used in the main manuscript.
**Figure S2:** WHAM‐reweighted hydrogen‐bond distance and angle distributions for water contacts to the thiocarbonyl sulfur in the H‐bond‐“off” system. The transition‐state region shows a reduced contact population, consistent with the transient S–C interaction discussed in the main manuscript, but no evidence for systematically shortened hydrogen bonds.

## Data Availability

The data that support the findings of this study are available from the corresponding author upon reasonable request.
