# Supplementary material for: On the Role of Hydrogen Bond Strength and Charge Transfer in an On‐Water Diels–Alder Reaction: Semiempirical and Free Energy Calculations
Source: J Comput Chem. 2026 Jul 20;47(20):e70467. doi: 10.1002/jcc.70467 (PMC13383029; doi:10.1002/jcc.70467)
Supplement: Supplementary file 1 — Figure S1: WHAM‐reweighted hydrogen‐bond distance and angle distributions for water contacts to the carbonyl oxygen in the H‐bond‐“on” system. The distributions compare representative reactant, transition‐state, and product regions along the same reaction coordinate used in the main manuscript. Figure S2: WHAM‐reweighted hydrogen‐bond distance and angle distributions for water contacts to the thiocarbonyl sulfur in the H‐bond‐“off” system. The transition‐state region shows a reduced contact population, consistent with the transient S–C interaction discussed in the main manuscript, but no evidence for systematically shortened hydrogen bonds. [file JCC-47-0-s001.pdf]

Received: Added at production | Revised: Added at production | Accepted: Added at production

DOI: xxx/xxxx

SUPPORTING INFORMATION

On the role of hydrogen bond strength and charge transfer in an on-water Diels–Alder reaction: Semiempirical and free energy calculations

Andrés Henao[1] | **Yomna Gohar[1]** | **René Wilhelm[2]** | **Thomas D. Kühne[3,4,5]**

[1]Dynamics of Condensed Matter, Chair of Theoretical Chemistry, Paderborn University, Warburger Str. 100, D-33098 Paderborn, Germany

[2]Institute of Organic Chemistry, Clausthal University of Technology, Leibnizstr. 6, D-38678 Clausthal-Zellerfeld, Germany

[3]Center for Advanced Systems Understanding (CASUS), Conrad-Schiedt-Straße 20, 02826 Görlitz, Germany

[4]Helmholtz Zentrum Dresden-Rossendorf, Bautzner Landstraße 400, 01328 Dresden, Germany

[5]Institute of Artificial Intelligence, Technische Universität Dresden, Helmholtzstraße 10, 01069 Dresden, Germany

Correspondence

Thomas D. Kühne, Center for Advanced Systems Understanding (CASUS), Conrad-Schiedt-Straße 20, 02826 Görlitz, Germany.
Email: tkuehne@cp2k.org

Present address

Yomna Gohar: International Max Planck Research School, Georg-August-Universität Göttingen, Wilhelmsplatz 1, 37073 Göttingen, Germany.

Summary

This Supporting Information provides WHAM-reweighted hydrogen-bond distance and angle distributions for the H-bond-“on" and H-bond-“off" systems discussed in the main manuscript.

HYDROGEN-BOND DISTANCE AND ANGLE DISTRIBUTIONS

The distributions shown below were obtained from the same WHAM-reweighted umbrella sampling windows used for the hydrogen-bond populations in Fig. 6 of the main manuscript. They are included to test whether the transition-state regions are associated with shorter or more directional hydrogen bonds. Within the statistical resolution of the present simulations, no systematic shortening of the hydrogen bonds is observed at the transition state.

 © 2026 Copyright Holder Name

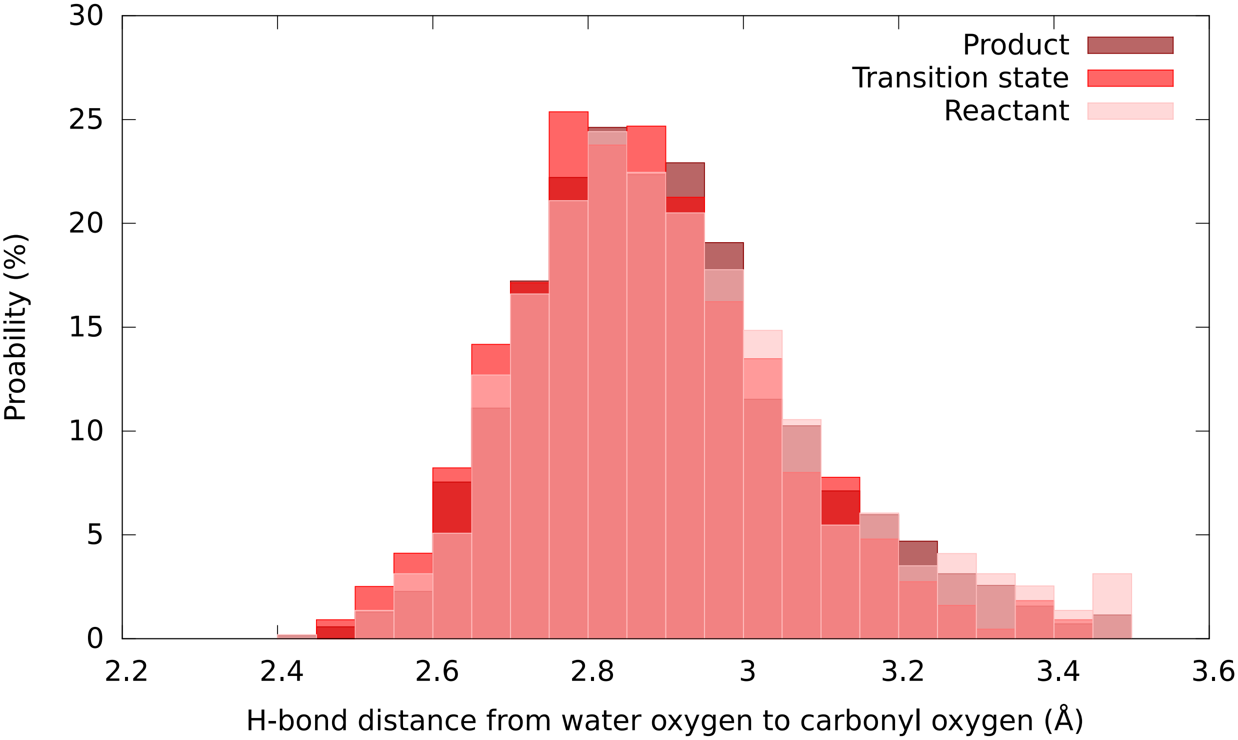

(a) H-bond-“on" distance distribution.

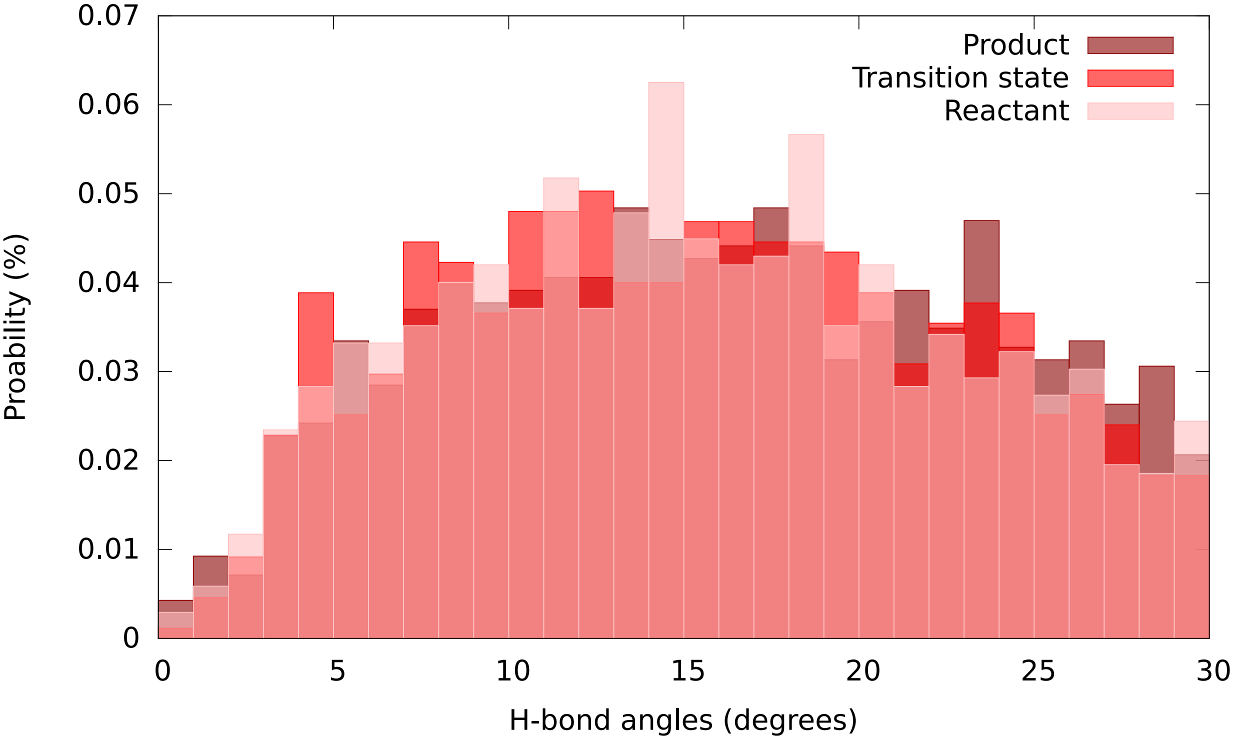

(b) H-bond-“on" angle distribution.

FIGURE S1 WHAM-reweighted hydrogen-bond distance and angle distributions for water contacts to the carbonyl oxygen in the H-bond-“on" system. The distributions compare representative reactant, transition-state, and product regions along the same reaction coordinate used in the main manuscript.

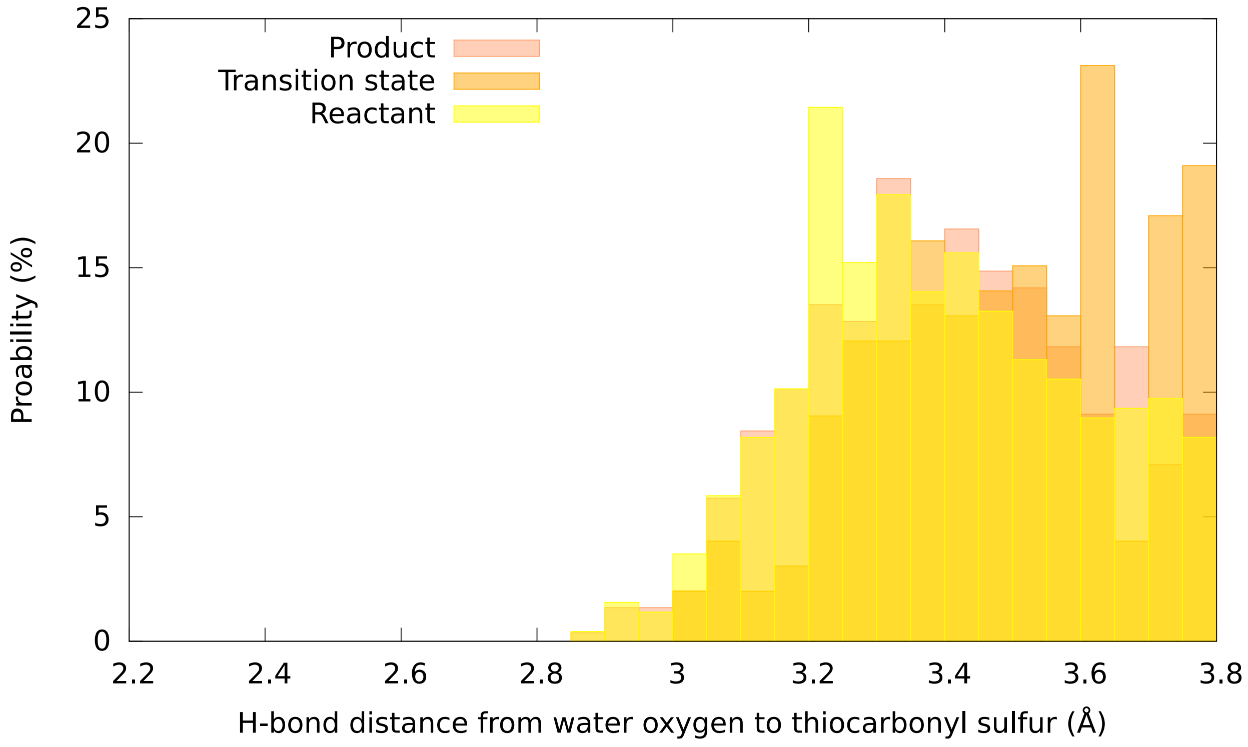

(a) H-bond-“off" distance distribution.

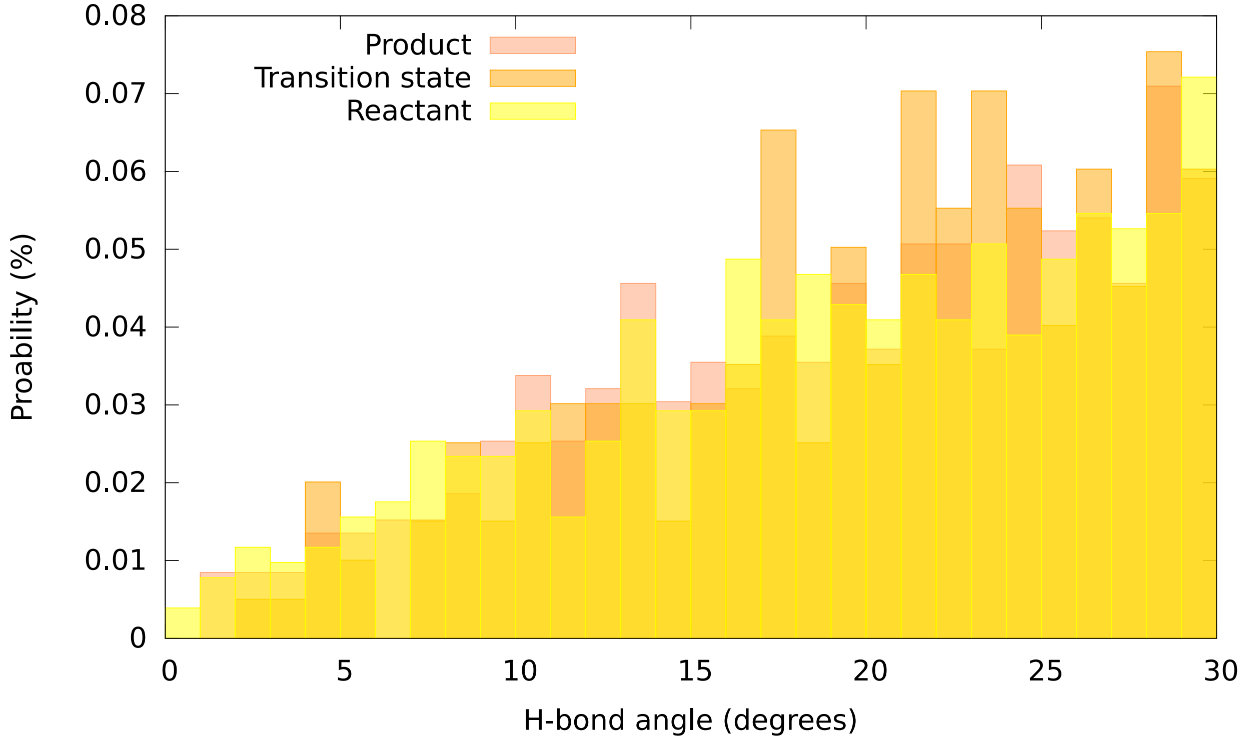

(b) H-bond-“off" angle distribution.

FIGURE S2 WHAM-reweighted hydrogen-bond distance and angle distributions for water contacts to the thiocarbonyl sulfur in the H-bond-“off" system. The transition-state region shows a reduced contact population, consistent with the transient S–C interaction discussed in the main manuscript, but no evidence for systematically shortened hydrogen bonds.
